# Linkage disequilibrium and within-breed genetic diversity in Iranian Zandi sheep

**DOI:** 10.5194/aab-62-143-2019

**Published:** 2019-04-02

**Authors:** Seyed Mohammad Ghoreishifar, Hossein Moradi-Shahrbabak, Nahid Parna, Pourya Davoudi, Majid Khansefid

**Affiliations:** 1 Department of Animal Science, University College of Agriculture and Natural Resources, University of Tehran, Karaj, 31587-11167, Iran; 2 Agriculture Victoria, AgriBio Centre for AgriBioscience, Bundoora, VIC 3083, Australia

## Abstract

This research aimed to measure the extent of linkage disequilibrium (LD), effective population size ($N_{e}$), and runs of homozygosity (ROHs) in one of the major Iranian sheep breeds (Zandi) using 96 samples genotyped with Illumina Ovine SNP50 BeadChip. The amount of LD ($r^{2}$) for single-nucleotide polymorphism (SNP) pairs in short distances (10–20 kb) was 0.21±0.25 but rapidly decreased to 0.10±0.16 by increasing the distance between SNP pairs (40–60 kb). The $N_{e}$ of Zandi sheep in past (approximately 3500 generations ago) and recent (five generations ago) populations was estimated to be 6475 and 122, respectively. The ROH-based inbreeding was 0.023. We found 558 ROH regions, of which 37 % were relatively long (>10 Mb). Compared with the rate of LD reduction in other species (e.g., cattle and pigs), in Zandi, it was reduced more rapidly by increasing the distance between SNP pairs. According to the LD pattern and high genetic diversity of Zandi sheep, we need to use an SNP panel with a higher density than Illumina Ovine SNP50 BeadChip for genomic selection and genome-wide association studies in this breed.

## 1 Introduction

The population of sheep in Iran, consisting of many different breeds, is estimated to be approximately 50 million, which constitutes the most important economic area in the livestock industry of the country (Moradi et al., 2012). According to Rashidi et al. (2008), the sheep industry contributes about 35 % of the meat production in Iran. As the supply of meat production from sheep does not satisfy the demand, a breeding plan seems reasonable to enhance the profitability of sheep production by improving such traits as litter size, body conformation, lamb weight, and other economically important traits (Ghafouri-Kesbi et al., 2008). The Zandi breed is one of the major, indigenous, fat-tailed sheep breeds of Iran that has adapted to survive in harsh weather conditions and poor and mountainous pastures in the central regions of the country (Mohammadi et al., 2013). Probably because of its large population in Iran (about 2 million heads), there is much interest in genetically improving Zandi sheep for growth traits (Ghafouri-Kesbi et al., 2008; Mohammadi et al., 2013).

The adoption of such new technologies as genomic selection can play an essential role in satisfying the demand for meat production. One advantage of genomic over traditional selection methods is that selection candidates can be evaluated more accurately at very early ages, which increases the rate of genetic gain (Hayes et al., 2013). Also, the accuracy of genomic selection is regularly higher than that of traditional pedigree-based selection (Goddard and Hayes, 2011). So, there is potential to increase genetic gain in Zandi sheep by adopting genomic selection technology; however, the success of genomic selection relies on the level of genome-wide linkage disequilibrium (LD) between markers and causal mutations (Hayes et al., 2013). The nonrandom association between alleles of different variants throughout the genome in the population is known as LD. The main reason for this association, or correlation between alleles of different markers, is their physical closeness. However, some other factors, such as genetic drift, migration, and, more importantly, either artificial or natural selection, also affect the LD and its pattern (Wang, 2005). Measuring the amount of genome-wide LD in a population can help us estimate the number of single-nucleotide polymorphism (SNP) markers required to have robust genomic evaluations (Hayes and Goddard, 2001) and genome-wide association studies (GWASs) (Carlson et al., 2004).

The extent of genome-wide LD can also help us estimate past effective population sizes ($N_{e}$) and rates of recombination in a population. Information on past $N_{e}$ is critical to define optimal selection pressures (Rexroad and Vallejo, 2009) for obtaining breeding goals while sustaining acceptable levels of genetic heterogeneity in breeding populations (Scraggs et al., 2014). Reduction in genetic diversity can reduce the profitability of animal production, which is often referred to as the “inbreeding effect” (Leroy, 2014; Notter, 1999). A meta-analysis of 57 studies on seven livestock species and a wide range of traits showed that approximately 0.14 % of the mean of the traits decreased for every 1 % increase in inbreeding (Leroy, 2014). The inbreeding coefficient can be investigated by studying the length of identical haplotype segments inherited from parents to progeny. In such investigations, the successive homozygous genotypes in the progeny are known as runs of homozygosity (ROHs). Hence, the aims of this research were to (1) measure the amount and pattern of LD, (2) examine genetic diversity by measuring $N_{e}$ and ROH-based inbreeding, and (3) estimate the minimum number of SNPs required for robust genomic selection and GWASs in Zandi sheep.

## 2 Materials and methods

### 2.1 Sample collection and quality control

Blood samples were taken from 99 Zandi sheep at the Khojir sheep breeding station in Iran. All experimental procedures were reviewed and approved by the research ethics committee of the University of Tehran, College of Agriculture and Natural Resources. The genomic DNA was extracted using the standard salting-out protocol (Helms, 1990). The samples were genotyped using Illumina Ovine SNP50 BeadChip for 54 241 SNP markers; the version of the ovine assembly used in our research was Ovis_aries_v4.0 obtained from the SNPchiMP v3 database (Nicolazzi et al., 2015). The filtering process was completed using PLINK V1.9 (Chang et al., 2015). Of 99 genotyped animals, three samples were excluded due to low genotype quality (missing genotypes >10 %), and the remaining 96 samples had a genotyping call rate >98.2 %. SNPs were removed due to (1) having an unknown map position (n=387), (2) being located on sex chromosomes (n=1449), (3) having an SNP missing rate of more than 10 % (n=948), (4) deviating from Hardy–Weinberg equilibrium (HWE) with a p value $<\;10^{-6}$ (n=132), and (5) having a minor allele frequency (MAF) of less than 0.01 (n=2068). Then, using a genomic relationship matrix (VanRaden, 2008), we ran a principal component analysis (PCA) to explore the covariance between animals.

### 2.2 LD calculation

LD was calculated by $r^{2}$ (Hill and Robertson, 1968) for two loci (A and B) with two alleles (allele 1 and allele 2) using Eq. (1):

1 $$r^{2}=\frac{\left\{\left[f\left(A_{1}B_{1}\right)\;f\left(A_{2}B_{2}\right)\right]-\left[f\left(A_{1}B_{2}\right)\;f\left(A_{2}B_{1}\right)\right]\;\right\}{}^{2}}{f\left(A_{1}\right)\;f\left(B_{1}\right)\;f\left(A_{2}\right)\;f\left(B_{2}\right)},$$

where $f\left(A_{1}B_{1}\right)$, $f\left(A_{1}B_{2}\right)$, $f\left(A_{2}B_{1}\right)$, and $f\left(A_{2}B_{2}\right)$ are the frequencies of haplotypes $A_{1}B_{1}$, $A_{1}B_{2}$, $A_{2}B_{1}$, and $A_{2}B_{2}$; $f\left(A_{1}\right)$, $\;f\left(A_{2}\right)$, $f\left(B_{1}\right)$, and $f(B_{2})$ are the frequencies of the first and second allele of loci A and B. The expected frequency for haplotype $A_{1}B_{1}$ is $f\left(A_{1}\right)f\left(B_{1}\right)$. Thus, the difference between the observed and expected frequency of a given haplotype indicates the LD between the two markers.

To measure LD, haplotypes were inferred using Beagle v3.3.2 (Browning and Browning, 2007), separately for each ovine autosome. Missing genotypes after quality control (0.018 %) were also imputed during haplotype phasing. The phased haplotypes were read into HAPLOVIEW v4.2 (Barrett et al., 2004) to calculate LD. For each autosome, $r^{2}$ was computed for all pairs of SNPs located up to 5 Mb apart. The pairwise LDs calculated by HAPLOVIEW were assigned into different classes according to pairwise distances (i.e., 0–10, 10–20, 20–40, 40–60, 60–100, 100–200, and 200–500 kb; 0.5–1.0, 1.0–2.0, and 2.0–5.0 Mb), and the average $r^{2}$ was calculated in each category using R software (R Core Team, 2014).

### 2.3 Estimation of $N_{e}$

The $N_{e}$ was estimated using SNeP v1.1 (Barbato et al., 2015) using Eq. (2) (Corbin et al., 2012):

2 $$N_{e\left(t\right)}=\left[\frac{1}{4f\left(c_{t}\right)}\right]\left[\frac{1}{E\left(r_{\mathrm{adjusted}}^{2}|c_{t}\right)}-\alpha \right],$$

where $N_{e\left(t\right)}$ is an estimation of $N_{e}$ t generations ago; $c_{t}$ is the pairwise genomic physical distance showing the recombination rate t generations ago (i.e., t=1/2c); and α is a constant in the equation to correct for the occurrence of mutations (if required). However, instead of assuming 1 cM =1 Mb, a recombination rate modifier in Eq. (3) was used to calculate c (Sved, 1971):

3 $$c=d\frac{1-(d/2)}{(1-d{)}^{2}},$$

where d is the linkage at distance c, which can be estimated using $r^{2}$ adjusted for sample size (adjustment required if the sample size is small). In this study, the default parameters of “1” and “no correction” were used for α and sample size adjustment, respectively.

### 2.4 ROH mapping

ROHs in the genotyped sheep were found using PLINK V1.9 according to the following criteria. Each ROH had at least one SNP per 100 kb and a minimum length of 4 Mb. The sliding window under examination was allowed to contain up to one heterozygous SNP, and the minimum number of SNPs for a given ROH was determined using Eq. (4) (Lencz et al., 2007):

4 $$l=\frac{\log_{e}\frac{\alpha }{n_{i}n_{s}}}{\log_{e}(1-\mathrm{het})},$$

where $n_{s}$ and $n_{i}$ represent the number of genotyped SNPs per animal and the number of animals, respectively; α is the percentage of false positive ROHs (set to 0.05 in the current study), and “het” is the mean SNP heterozygosity across all SNPs. Using R software (R Core Team, 2014), the detected ROH was assigned into different length categories, including 4–10, 10–20, 20–30, and >30 Mb, and the frequency of different ROH lengths was calculated. Moreover, ROH-based inbreeding ($F_{\mathrm{ROH}}$) for each individual was calculated using Eq. (5) (McQuillan et al., 2008):

5 $$F_{{\mathrm{ROH}}_{i}}=\frac{\sum_{j=1}^{n}L_{{\mathrm{ROH}}_{j}}}{L_{\mathrm{Genome}}}$$

where $F_{{\mathrm{ROH}}_{i}}$ is the $F_{\mathrm{ROH}}$ (inbreeding) for the ith animal; n and $L_{{\mathrm{ROH}}_{j}}$ are the total number of ROHs and the length of the jth ROH for the ith animal, respectively; and $L_{\mathrm{Genome}}$ is the genome length covered by the SNP markers (i.e., 2645.2 Mb in our study).

## 3 Results

### 3.1 Descriptive statistics

**Figure 1:**
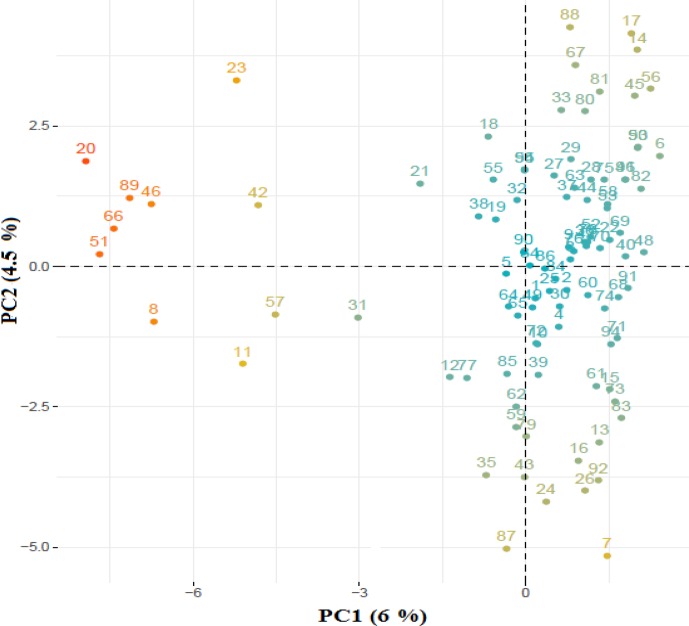
PCA plot based on a genomic relationship matrix. The first two principal components (PCs) and the variance explained by each component is shown on the corresponding axis. The amount of variance explained by the first two components is relatively small (10.5 %), representing a great deal of genetic diversity among samples.

After quality control, the final dataset consisted of 96 animals genotyped for 49 257 SNPs covering 2.6452 Gb of the ovine genome. The average distance between adjacent SNPs was 53.73±55.60 kb, and the average MAF was 0.28±0.01. The number of SNPs varied on ovine autosomes, with OAR1 as the longest and OAR24 as the shortest ovine autosome containing 11.3 % (n=5563) and 1.4 % (n=702) of SNPs, respectively. The average inter-marker distance was the shortest in OAR9 (49.9 kb) and the longest in OAR21 (65.2 kb). Further details for the SNPs located on the same chromosome are presented in Table 1. The distribution of MAF for the SNPs that passed the quality control process is shown as a histogram in Supplement Fig. S1; approximately 48 % of the SNPs had MAF ≥0.30 and approximately 5.1 % of the SNPs had MAF between 0.01 and 0.05. As illustrated in Fig. 1, the relationship between the first two principal components described only 10.5 % of the total variation among samples.

**Table 1:** Summary statistics of single-nucleotide polymorphism (SNP) markers for each Ovine autosome.

OAR${}^{*}$	Number	OAR length	Distance between adjacent markers (kb)			
	of SNPs	(Mb)	Average	SD	Median	Maximum
1	5563	299.6	53.86	49.81	44.31	913.19
2	5183	263.1	50.77	42.36	43.09	898.52
3	4678	242.7	51.89	47.83	43.00	1335.67
4	2520	127.1	50.46	37.72	43.62	474.43
5	2225	116.0	52.14	46.70	43.65	842.19
6	2439	129.0	52.92	74.23	42.54	2936.69
7	2115	108.7	51.41	49.14	43.34	1053.01
8	1937	97.8	50.51	41.06	42.62	463.76
9	2020	100.8	49.92	41.02	42.15	789.82
10	1717	94.1	54.84	112.45	43.05	3418.84
11	1110	66.9	60.29	52.83	48.92	477.63
12	1606	85.8	53.49	56.02	43.47	1167.57
13	1603	88.9	55.47	49.42	45.92	903.00
14	1093	68.7	62.96	69.38	48.12	1328.81
15	1581	89.8	56.86	65.97	45.03	1631.39
16	1488	77.1	51.83	39.67	43.29	424.04
17	1340	78.4	58.58	48.44	47.96	562.71
18	1328	71.9	54.17	45.48	45.18	538.75
19	1165	64.8	55.67	42.58	46.03	416.87
20	1071	55.5	51.90	51.52	42.77	1044.18
21	846	55.1	65.25	98.90	49.75	2422.03
22	1026	55.0	53.68	82.14	43.52	2257.62
23	1075	66.3	61.72	51.34	48.55	715.16
24	702	44.2	63.10	45.65	52.15	343.63
25	952	48.0	50.52	39.50	43.68	589.56
26	874	49.8	57.07	69.51	46.65	1754.10

${}^{*}$ *Ovis aries* autosome.

### 3.2 Extent of genome-wide LD

The reduction in LD ($r^{2}$) by increasing the pairwise distance between SNPs is presented in Table 2. The average $r^{2}$ was 0.26±0.30 when the pairwise distance between SNPs was <10 kb, but it rapidly decreased to 0.21±0.25 when the distance increased to 10–20 kb. At the pairwise distance of 40–60 kb, which was close to the average inter-marker space in this study, the average $r^{2}$ was only 0.10±0.16. The percentage of pairwise SNPs with high LD ($r^{2}\geq 0.20$) up to 10 kb apart was 39 %, which decreased to 16 % for the SNP pairs with 40–60 kb of distance (Table 2). The average LD between SNPs with up to 5 Mb of distance (4 481 704 SNP pairs) is presented for all 26 ovine autosomes (Table S1 in the Supplement). Moreover, $r^{2}$ was calculated for only the adjacent SNP pairs (0.13±0.19), of which up to 51 % of SNP pairs had $r^{2}<0.05$ and only 19.1 % had $r^{2}>0.2$ (Fig. S2).

**Table 2:** Summary statistics of average of $r^{2}$ calculated for each distance category.

Distance	Number of	$r^{2}$			
(Mb)	SNP pairs	Average (±SD)	Median	Proportion of	Proportion of
				$r^{2}>0.2$	$r^{2}>0.3$
0.00–0.01	1998	0.26±0.30	0.13	0.39	0.302
0.01–0.02	4668	0.21±0.25	0.1	0.332	0.245
0.02–0.04	16 029	0.14±0.20	0.06	0.218	0.143
0.04–0.06	19 595	0.10±0.16	0.04	0.156	0.092
0.06–0.10	38 188	0.08±0.13	0.03	0.099	0.055
0.10–0.20	94 912	0.05±0.08	0.02	0.048	0.022
0.20–0.50	279 781	0.04±0.06	0.02	0.286	0.082
0.50–1.00	460 532	0.03±0.05	0.02	0.013	0.002
1.00–2.00	909 607	0.03±0.04	0.02	0.01	0.002
2.00–5.00	2 656 394	0.03±0.04	0.01	0.006	0.001

### 3.3 $N_{e}$

**Figure 2:**
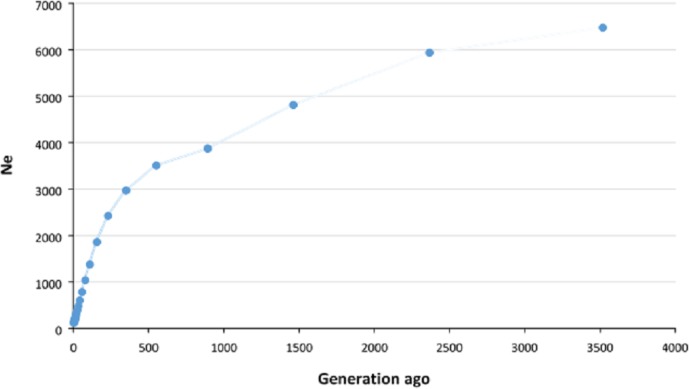
Estimation of effective population size ($N_{e}$) of Zandi sheep using genome-wide linkage disequilibrium ($r^{2}$) between SNPs. $N_{e}$ was estimated using average $r^{2}$ between markers at different distances by SNeP software. The $N_{e}$ in the past (approximately 3500 generations ago) was 6475, which decreased to 3000 approximately 500 generations ago and then continued to decrease more rapidly until recent generations. The $N_{e}$ of recent populations (up to five generations ago) was estimated to be 122.

The historical and recent $N_{e}$ values were estimated using average $r^{2}$ calculated in different pairwise distances between SNPs. The $N_{e}$ of approximately 3500 generations ago was estimated to be 6475, which decreased to approximately 3000 about 500 generations ago and then continued to decline more rapidly until recent generations. We estimated that the $N_{e}$ for Zandi sheep five generations ago was 122 (Fig. 2). A plot magnifying the changes in $N_{e}$ of more recent generations is also illustrated in Fig. S3.

### 3.4 ROHs and ROH-based inbreeding ($F_{\mathrm{ROH}}$)

**Figure 3:**
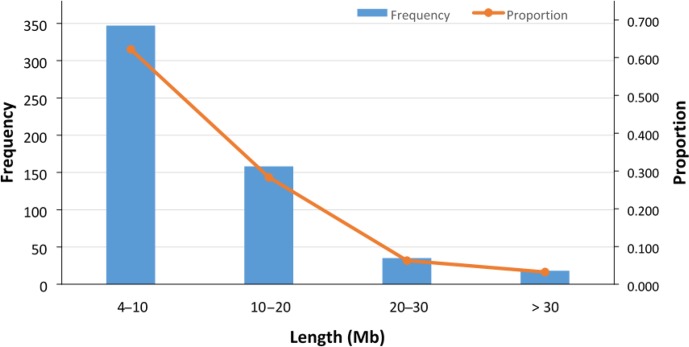
Frequency and proportion of detected ROHs with different lengths (Mb). In total, 558 ROHs (with average length of 10.77±8.36 Mb) were detected in 89 sheep, and in 7 remaining sheep no ROHs were found. The ROH segments with the length of 4–10 Mb were the most frequent (62.2 %), and those with the length of >30 Mb were the least frequent (3.2 %).

In total, 558 ROHs were detected for 89 sheep with an average length of 10.77±8.36 Mb (Fig. 3), and no ROH was found for the 7 remaining sheep (Table S2). The ROH segments with a length of 4–10 Mb were the most frequent (62 %), and those with a length of more than 30 Mb were the least frequent (3.2 %) (Fig. 3). The average number and median of ROHs detected in the 89 sheep were 6.3±4.2 and 5, respectively. The maximum number of ROHs found in one animal was 19. The longest ROH (72.4 Mb) consisted of 1425 SNPs, and the shortest ROH (4.02 Mb) consisted of 64 SNPs (Table S2).

**Figure 4:**
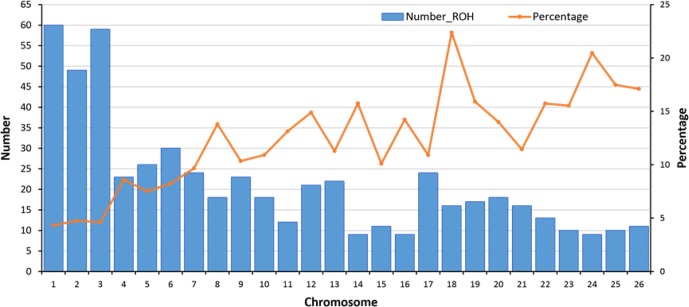
Number of detected ROHs and percent of coverage per autosome. In total, 558 ROHs were detected. For each autosome, the average length of a ROH (for the sheep with at least one ROH) was divided by the length of the chromosome to calculate the percentage of each autosome covered by ROHs. OAR1 with 60 ROHs (average length of 12.9±8.8 Mb) had the highest number of ROHs, and OAR14, OAR16, and OAR24 with 9 ROHs (average length of 10.3±5.9 Mb) had the lowest number of ROHs.

In ovine autosomes of Zandi sheep in our study, OAR1 had 60 ROHs, but OAR14, 16, and 24 had only 9 ROHs (Fig. 4). Hence, these chromosomes had the most and the minimum number of ROHs, respectively. We found a strong correlation (Pearson's correlation coefficient +0.95) between the number of ROHs and chromosome length and also between percentage of coverage and chromosome length (Pearson's correlation coefficient -0.79) (see Table 1 and Fig. 4). The average of ROH-based inbreeding ($F_{\mathrm{ROH}}$) for Zandi sheep with and without including the seven animals that had no ROHs was 0.023 and 0.026, respectively. The four animals with the highest degree of homozygosity indicated 302.4, 275.1, 234.9, and 178.4 Mb of their genome classified as ROHs, covering close to 9 % of their total genome length (Table S2).

## 4 Discussion

One of the main factors affecting LD measurement is population structure. Overestimation of LD occurs when samples are close relatives; in fact, they share longer haplotypes than distant relatives (Ghoreishifar et al., 2018; Gusev et al., 2011). In the Zandi breed, LD remained at a moderate level ($r^{2}=0.26\pm 0.30$) only up to 10 kb and decreased rapidly to 0.10±0.16 when the average SNP pair distance was 40–60 kb. This pattern of LD decay illustrates that short-length haplotypes are prevalent in the population, which means that samples are not close relatives. Also, only 10.5 % of the total variation is explained by the first two principal components, representing a great deal of diversity among samples.

The average of $r^{2}$ varied across autosomes (ranging from 0.16 in OAR26 to 0.34 in OAR10 for the pairwise distance of <10 kb), which is in line with previous reports in sheep (Liu et al., 2017; Prieur et al., 2017; Zhao et al., 2014), beef cattle (Bohmanova et al., 2010; Edea et al., 2015), and dairy cattle (Qanbari et al., 2010). This phenomenon can be due to variation in the recombination rate in different autosomes, natural or artificial selection, and genetic drift (Liu et al., 2017; Mastrangelo et al., 2017; Qanbari et al., 2010). Moreover, the variation in $r^{2}$ estimated for different chromosomes was higher in short SNP pair distances (e.g., SD =0.048 in 0–10 kb vs. SD =0.001 in 4.0–5.0 Mb), which is in line with the results reported by Liu et al. (2017).

The $r^{2}$ we calculated for the Zandi breed was close to the amount of LD reported in some other sheep breeds. For example, in Churra sheep, the average $r^{2}$ for the pairwise space of 0–10 kb and 0.5–1.0 Mb was 0.33 and 0.05, respectively (García-Gámez et al., 2012). In Chinese Merino sheep, the average $r^{2}$ for the same marker intervals was 0.25 and 0.02, respectively (Liu et al., 2017). However, in Barbaresca sheep, with a small sample size, the average $r^{2}$ for the inter-marker distance of 0.5–1.0 Mb was 0.12 (Mastrangelo et al., 2017). The average $r^{2}$ for SNPs within 10 kb of distance for Australian Border Leicester, Poll Dorset, and Merino sheep breeds were 0.34, 0.33, and 0.27, respectively (Al-Mamun et al., 2015). Thus, the variation in the reported $r^{2}$ in different breeds suggests that the characteristics of LD are highly breed-specific in sheep.

The power of quantitative trait locus (QTL) mapping and the accuracy of genomic predictions largely depend on the amount of LD between SNPs and quantitative trait nucleotides (QTNs) (Hayes et al., 2013). Generally, when the extent of LD between markers decreases rapidly by increasing the inter-marker distances, the number of markers required for a robust genomic study should be kept high. Hayes and Goddard (2001) demonstrated that the accuracy of genomic predictions for dairy cattle reached 85 % when the average $r^{2}$ between adjacent markers was ≥0.2. However, higher LD between markers ($r^{2}\geq 0.3$) might be required for precise QTL mapping (Corbin et al., 2012). In our study, the average $r^{2}$ was 0.21±0.25 when the distance between pairwise SNPs was 10–20 kb. However, the average inter-marker distance was 53.73±55.60 kb, and the average $r^{2}$ for only adjacent SNPs was 0.13±0.19 (19 % of adjacent SNPs had $r^{2}>0.2$). Hence, our findings support the necessity of denser SNP panels for a successful genomic selection scheme in Zandi sheep.

The level of LD in Zandi sheep was less than it is in cattle (Biegelmeyer et al., 2016; Bohmanova et al., 2010; Jasielczuk et al., 2016) and pigs (Grossi et al., 2017) but close to the level of LD reported for other sheep breeds (Al-Mamun et al., 2015; García-Gámez et al., 2012; Liu et al., 2017). For example, Qanbari et al. (2010) reported $r^{2}$ of 0.20±0.24 for SNPs within 50–75 kb in German Holstein dairy cattle. Moreover, the average $r^{2}$ for inter-marker distance <10 kb in Hereford and Bradford beef cattle breeds was reported at 0.49 and 0.43, respectively (Biegelmeyer et al., 2016). We found that for a very short inter-marker distance (<10 kb), the calculated $r^{2}$ in Zandi sheep was 0.26±0.30.

For the very recent generations (i.e., fewer than five generations ago), the accuracy of estimating $N_{e}$ is negatively affected by the low level of LD between SNPs at long distances (Liu et al., 2017). Thus, we estimated the $N_{e}$ in Zandi sheep up to five generations ago. The estimated $N_{e}$ in Zandi sheep for 59 and 80 generations ago was 786 and 1035, respectively, which is in line with the large $N_{e}$ (>500) reported for various breeds of sheep in the HapMap project (Kijas et al., 2012). The low level of $r^{2}$ even at relatively short distances shows the $N_{e}$ in Zandi sheep was large in recent past generations compared with other species. For example, the $r^{2}$ for SNP pairs within 0.9–1.0 Mb and the $N_{e}$ in recent generations in Duroc pigs were reported to be 0.2 and 75, respectively (Grossi et al., 2017). The $r^{2}$ (same SNP pair distance) and the $N_{e}$ in recent generations of Zandi sheep were 0.07 and 122. The estimated genome-based $N_{e}$ (i.e., $N_{e\;\mathrm{SNP}}=122$) in the current study was higher than the pedigree-based $N_{e}$ reported for Zandi (i.e., $N_{e\;\mathrm{PED}}=66$) in Ghafouri-Kesbi et al. (2008), in which the estimated $N_{e\;\mathrm{PED}}$ is likely to be less accurate due to the lack of precise and in-depth pedigree records for this breed. However, results should be interpreted with caution since other factors such as sampling itself, for example, can potentially affect the results. The threshold of 100 for $N_{e}$ has been recommended for many species to maintain genetic diversity in the population (Meuwissen, 2009). The $N_{e}$ of Zandi sheep five generations ago (122) was above the recommended critical threshold. However, given the sharp drop in $N_{e}$ in recent generations, we should be careful to maintain the $N_{e}$ larger than 100.

We observed that the $N_{e}$ decreased more intensively from roughly 550 generations ago, which is in agreement with the results reported for a couple of Iranian fat-tailed sheep breeds by Moradi et al. (2017). Considering a generation interval of 4 to 5 years for sheep, this point coincides with the period when the first archaeological evidence of Iranian fat-tailed sheep breeds at Takht-Jamshid, Iran, was obtained (Moradi et al., 2017). These results may support the hypothesis that selection for fat-tailed trait in Iranian indigenous sheep populations occurred nearly 2500 years ago and has continued since then. However, more evidence is required to reinforce this hypothesis.

One of the main factors affecting autozygosity estimates is the density of SNP chips applied to generate the data for ROH studies. Marras et al. (2015), in a study of ROHs applying a medium-density chip, reported that when heterozygous SNPs were allowed, the number of longer ROHs increased dramatically and suggested not using them in the ROH. Ferenčaković et al. (2013) reported that the 50K SNP panel led to the overestimation of short-length ROHs (<4 Mb), probably because of heterozygous SNPs on high-density chips that may not exist on a medium-density one. Hence, they proposed that the 50K panel does not have enough sensitivity for the accurate determination of short runs of homozygosity (i.e., <4 Mb length). In our study, because a medium-density SNP panel was adopted, we outlined ROHs as regions of homozygous genotypes that had at least 4 Mb of length distinguished with a maximum number of one heterozygous SNP.

Long ROHs in an individual can occur due to either inbreeding events or selection pressure (Mastrangelo et al., 2016). Since the Zandi population is subject to selection programs (Ghafouri-Kesbi et al., 2008; Mohammadi et al., 2013), the occurrence or runs of homozygosity in this breed can be attributed to both inbreeding effects and selection pressure. In the ROH analysis of Zandi sheep, around 37.8 % of the detected ROH was relatively long (>10 Mb) and 62.2 % was shorter than 10 Mb. As reported in the literature, in Barbaresca sheep, 67 % of the detected ROH was short (ranging from 1 to 10 Mb) (Mastrangelo et al., 2017), and in some local breeds of dairy cattle, approximately 23 % of the detected ROH was longer than 10 Mb (Mastrangelo et al., 2016). However, comparing different ROH studies is not a straightforward matter because different researchers use different criteria, especially for the minimum length of a ROH and the minimum number of SNPs included in a ROH.

## 5 Conclusions

We found that in Zandi sheep, the amount of LD was relatively small between the adjacent SNPs in Illumina Ovine SNP50 BeadChip (0.13±0.19), and it decreased rapidly by increasing the distance between the markers. Therefore, a high-density SNP panel is required for a robust genomic selection and fine mapping of QTLs in this breed. Given the total length of autosomes in sheep (2645.1 Gb) and the average level of LD between SNPs in 5 kb ($r^{2}\approx 0.26$) and 15 kb ($r^{2}\approx 0.21$), we estimated that approximately 180 000 and 530 000 evenly spaced SNPs are required to increase the $r^{2}$ to 0.20 and 0.25, respectively. Compared with other species, average inbreeding in Zandi sheep was found to be relatively low (0.023). In our study, 7 sheep were not inbred ($F_{\mathrm{ROH}}=0$), and the inbreeding level of 35 sheep was more than the average level of inbreeding. Thus, optimal mating designs can be beneficial in controlling population inbreeding in Zandi sheep. Although the $N_{e}$ of the Zandi breed is not critically low, given the sharp decrease in $N_{e}$ in recent generations, we should be careful to ensure the $N_{e}$ remains large.

## Supplement

10.5194/aab-62-143-2019-supplement The supplement related to this article is available online at: https://doi.org/10.5194/aab-62-143-2019-supplement.

## Data Availability

The original data are available upon request to the corresponding authors.
