# Supplementary material for: Linkage disequilibrium and within-breed genetic diversity in Iranian Zandi sheep
Source: Arch Anim Breed. 2019 Apr 2;62(1):143–51. doi: 10.5194/aab-62-143-2019 (PMC6852851; doi:10.5194/aab-62-143-2019)
Supplement: The supplement related to this article is available online at: https://doi.org/10.5194/aab-62-143-2019-supplement. [file aab-62-143-supplement.pdf]

Supplement of Arch. Anim. Breed., 62, 143–151, 2019
https://doi.org/10.5194/aab-62-143-2019-supplement
© Author(s) 2019. This work is distributed under
the Creative Commons Attribution 4.0 License.

Open Access
Archives Animal Breeding

Supplement of

Linkage disequilibrium and within-breed genetic diversity in Iranian Zandi sheep

Seyed Mohammad Ghoreishifar et al.

Correspondence to: Hossein Moradi-Shahrbabak (hmoradis@ut.ac.ir)

The copyright of individual parts of the supplement might differ from the CC BY 4.0 License.

Table S1. Average linkage disequilibrium (r^2) calculated in each ovine autosome for the single-nucleotide polymorphism (SNP) with different pairwise distance

	Pairwise distance (kb)											Pairwise distance (Mb)				
OAR	0-10	10-20	20-30	30-40	40-50	50-60	60-70	70-80	80-90	90-100	100-500	0.5-1.0	1.0-2.0	2.0-3.0	3.0-4.0	4.0-5.0
1	0.270	0.215	0.163	0.127	0.119	0.100	0.088	0.072	0.073	0.071	0.043	0.035	0.033	0.030	0.029	0.027
2	0.196	0.175	0.159	0.141	0.128	0.116	0.106	0.085	0.079	0.077	0.044	0.034	0.033	0.030	0.029	0.027
3	0.256	0.206	0.143	0.135	0.123	0.099	0.096	0.077	0.076	0.066	0.041	0.034	0.031	0.029	0.028	0.026
4	0.279	0.184	0.158	0.126	0.120	0.095	0.099	0.089	0.075	0.069	0.041	0.034	0.031	0.029	0.027	0.025
5	0.286	0.217	0.151	0.123	0.102	0.100	0.079	0.083	0.071	0.070	0.042	0.035	0.033	0.031	0.029	0.027
6	0.220	0.198	0.164	0.121	0.104	0.095	0.081	0.073	0.067	0.067	0.043	0.034	0.032	0.030	0.028	0.026
7	0.278	0.224	0.157	0.134	0.107	0.108	0.082	0.075	0.072	0.069	0.041	0.035	0.032	0.030	0.028	0.025
8	0.334	0.238	0.168	0.132	0.103	0.084	0.084	0.069	0.061	0.066	0.040	0.035	0.033	0.030	0.028	0.027
9	0.259	0.233	0.149	0.115	0.106	0.102	0.085	0.084	0.074	0.062	0.042	0.035	0.032	0.030	0.028	0.026
10	0.341	0.239	0.168	0.129	0.117	0.109	0.116	0.086	0.082	0.073	0.049	0.038	0.033	0.031	0.029	0.026
11	0.262	0.221	0.135	0.138	0.089	0.086	0.087	0.066	0.064	0.063	0.038	0.031	0.028	0.027	0.026	0.024
12	0.274	0.187	0.137	0.125	0.107	0.093	0.087	0.067	0.071	0.067	0.042	0.035	0.033	0.030	0.028	0.026
13	0.248	0.236	0.170	0.142	0.112	0.110	0.096	0.090	0.082	0.075	0.044	0.036	0.034	0.031	0.029	0.027
14	0.221	0.204	0.129	0.102	0.117	0.089	0.093	0.055	0.069	0.071	0.036	0.030	0.028	0.026	0.024	0.022
15	0.266	0.202	0.124	0.128	0.105	0.102	0.082	0.079	0.071	0.067	0.041	0.034	0.032	0.029	0.027	0.025
16	0.229	0.204	0.140	0.107	0.104	0.086	0.078	0.073	0.064	0.067	0.038	0.033	0.031	0.029	0.028	0.025
17	0.242	0.237	0.146	0.135	0.104	0.090	0.077	0.088	0.065	0.062	0.038	0.032	0.030	0.028	0.026	0.024
18	0.274	0.196	0.152	0.108	0.100	0.096	0.076	0.083	0.067	0.065	0.038	0.031	0.029	0.027	0.025	0.024
19	0.169	0.131	0.093	0.142	0.103	0.102	0.107	0.069	0.064	0.057	0.044	0.036	0.032	0.030	0.028	0.026
20	0.220	0.188	0.127	0.098	0.084	0.087	0.066	0.079	0.064	0.061	0.040	0.034	0.030	0.028	0.026	0.024
21	0.336	0.179	0.168	0.127	0.107	0.098	0.091	0.076	0.059	0.066	0.044	0.037	0.035	0.031	0.030	0.028
22	0.311	0.216	0.134	0.125	0.098	0.099	0.078	0.077	0.066	0.069	0.041	0.037	0.033	0.029	0.027	0.026
23	0.327	0.177	0.127	0.121	0.128	0.081	0.078	0.068	0.069	0.062	0.043	0.038	0.035	0.032	0.030	0.028
24	0.236	0.222	0.179	0.120	0.108	0.081	0.077	0.066	0.043	0.045	0.035	0.032	0.029	0.027	0.024	0.023
25	0.295	0.202	0.152	0.109	0.103	0.071	0.078	0.061	0.063	0.065	0.040	0.035	0.033	0.030	0.028	0.025
26	0.160	0.211	0.138	0.124	0.109	0.090	0.071	0.074	0.047	0.060	0.037	0.034	0.031	0.029	0.027	0.025

**Mean**	**0.259**	**0.205**	**0.150**	**0.127**	**0.112**	**0.099**	**0.089**	**0.077**	**0.071**	**0.068**	**0.042**	**0.035**	**0.032**	**0.030**	**0.028**	**0.026**
**SD**	**0.300**	**0.253**	**0.206**	**0.187**	**0.169**	**0.154**	**0.154**	**0.124**	**0.116**	**0.114**	**0.064**	**0.047**	**0.043**	**0.040**	**0.038**	**0.035**

Table S2. Detected runs of homozygosity (ROH) in Zandi sheep

Number	ID	Number of ROH	ROH Length (Mb)					FROH
			Average	Min.	Max.	Median	Total	
1	V_1_Plate1_A1	3	7.38	9.473	4.628	8.039	22.139	0.008
2	V_13_Plate1_B1	1	4.457	4.457	4.457	4.457	4.457	0.002
3	V_25_Plate1_C1	6	8.317	17.87	4.024	7.199	49.9	0.019
4	V_37_Plate1_D1	6	8.05	17.096	4.323	6.767	48.301	0.018
5	V_49_Plate1_E1	2	11.379	13.647	9.11	11.379	22.757	0.009
6	V_61_Plate1_F1	6	7.728	11.48	4.213	7.193	46.368	0.018
7	V_73_Plate1_G1	12	13.675	41.366	4.069	10.443	164.098	0.062
8	V_85_Plate1_H1	1	4.617	4.617	4.617	4.617	4.617	0.002
9	V_2_Plate1_A2	5	7.096	10.336	5.105	5.807	35.48	0.013
10	V_14_Plate1_B2	2	5.306	6.34	4.272	5.306	10.611	0.004
11	V_50_Plate1_E2	2	16.68	29.264	4.095	16.68	33.359	0.013
12	V_74_Plate1_G2	5	5.871	6.867	4.428	6.672	29.354	0.011
13	V_86_Plate1_H2	5	10.717	22.624	4.461	9.956	53.584	0.02
14	V_3_Plate1_A3	3	7.788	11.597	4.297	7.47	23.364	0.009
15	V_15_Plate1_B3	7	12.062	23.044	5.238	9.475	84.432	0.032
16	V_27_Plate1_C3	9	14.684	47.579	4.069	12.198	132.156	0.05
17	V_39_Plate1_D3	7	7.875	12.91	4.943	6.424	55.127	0.021
18	V_51_Plate1_E3	13	10.476	23.695	4.848	7.909	136.183	0.051
19	V_63_Plate1_F3	1	7.245	7.245	7.245	7.245	7.245	0.003
20	V_75_Plate1_G3	15	9.442	22.04	4.021	6.205	141.626	0.054
21	V_87_Plate1_H3	10	9.023	19.394	4.295	7.747	90.232	0.034
22	V_4_Plate1_A4	9	11.052	16.222	5.95	11.737	99.467	0.038
23	V_28_Plate1_C4	5	7.795	13.035	4.257	8.655	38.977	0.015
24	V_40_Plate1_D4	6	9.418	14.1	4.415	9.871	56.51	0.021
25	V_52_Plate1_E4	5	8.145	11.837	4.421	7.674	40.726	0.015
26	V_64_Plate1_F4	2	9.967	12.604	7.331	9.967	19.934	0.008
27	V_76_Plate1_G4	6	7.763	15.898	4.686	6.381	46.575	0.018
28	V_88_Plate1_H4	5	9.605	17.077	4.053	6.531	48.024	0.018
29	V_5_Plate1_A5	3	5.353	7.758	4.063	4.239	16.06	0.006
30	V_17_Plate1_B5	4	11.756	17.099	5.386	12.269	47.023	0.018
31	V_29_Plate1_C5	1	8.687	8.687	8.687	8.687	8.687	0.003
32	V_41_Plate1_D5	3	6.211	10.189	4.166	4.277	18.632	0.007
33	V_53_Plate1_E5	2	6.139	6.176	6.101	6.139	12.277	0.005
34	V_65_Plate1_F5	13	10.358	26.58	4.064	9.212	134.651	0.051
35	V_77_Plate1_G5	3	10.228	16.263	4.647	9.774	30.684	0.012
36	V_89_Plate1_H5	6	7.725	14.496	4.265	6.684	46.349	0.018
37	V_6_Plate1_A6	2	11.527	18.99	4.064	11.527	23.053	0.009
38	V_18_Plate1_B6	2	12.899	20.928	4.87	12.899	25.799	0.01
39	V_30_Plate1_C6	4	14.569	17.305	9.34	15.815	58.274	0.022

40	V_42_Plate1_D6	9	14.119	32.429	4.132	12.144	127.067	0.048
41	V_54_Plate1_E6	5	5.947	6.91	4.6	6.249	29.735	0.011
42	V_66_Plate1_F6	6	14.633	29.963	4.354	12.545	87.797	0.033
43	V_78_Plate1_G6	15	11.892	24.841	4.17	11.188	178.375	0.067
44	V_90_Plate1_H6	1	5.029	5.029	5.029	5.029	5.029	0.002
45	V_7_Plate1_A7	8	6.835	17.652	4.113	4.581	54.68	0.021
46	V_19_Plate1_B7	15	9.481	17.623	4.058	8.959	142.215	0.054
47	V_31_Plate1_C7	2	9.339	13.006	5.673	9.339	18.679	0.007
48	V_43_Plate1_D7	5	15.619	31.129	5.32	16.146	78.093	0.03
49	V_55_Plate1_E7	16	14.68	40.975	5.714	10.967	234.873	0.089
50	V_67_Plate1_F7	7	8.44	15.461	4.563	7.366	59.077	0.022
51	V_79_Plate1_G7	2	12.97	21.495	4.446	12.97	25.941	0.01
52	V_91_Plate1_H7	11	14.46	58.577	4.555	7.683	159.063	0.06
53	V_8_Plate1_A8	2	7.387	9.026	5.747	7.387	14.773	0.006
54	V_20_Plate1_B8	4	11.244	20.515	6.139	9.16	44.974	0.017
55	V_32_Plate1_C8	3	6.656	9.157	4.381	6.429	19.967	0.008
56	V_44_Plate1_D8	10	7.319	17.263	4.241	6.824	73.185	0.028
57	V_56_Plate1_E8	11	11.759	41.113	4.053	7.306	129.352	0.049
58	V_68_Plate1_F8	10	6.458	9.181	4.317	6.073	64.578	0.024
59	V_80_Plate1_G8	10	9.111	23.249	4.101	9.072	91.114	0.034
60	V_21_Plate1_B9	19	15.916	51.839	4.855	10.012	302.412	0.114
61	V_33_Plate1_C9	10	13.959	40.616	4.278	10.03	139.588	0.053
62	V_45_Plate1_D9	1	4.11	4.11	4.11	4.11	4.11	0.002
63	V_57_Plate1_E9	7	8.281	18.452	4.504	6.012	57.965	0.022
64	V_69_Plate1_F9	5	13.805	34.721	5.886	8.41	69.026	0.026
65	V_81_Plate1_G9	3	25.454	53.63	5.503	17.23	76.363	0.029
66	V_93_Plate1_H9	9	10.468	16.846	4.429	11.994	94.213	0.036
67	V_10_Plate1_A10	4	6.547	9.718	4.897	5.785	26.186	0.01
68	V_22_Plate1_B10	6	14.26	26.526	5.97	11.684	85.559	0.032
69	V_34_Plate1_C10	7	9.448	19.149	4.658	8.205	66.133	0.025
70	V_46_Plate1_D10	10	9.448	18.359	4.665	6.801	94.48	0.036
71	V_58_Plate1_E10	5	9.407	18.897	4.675	7.351	47.033	0.018
72	V_70_Plate1_F10	6	13.004	28.297	4.375	10.914	78.022	0.029
73	V_82_Plate1_G10	2	4.421	4.705	4.136	4.421	8.842	0.003
74	V_94_Plate1_H10	1	14.798	14.798	14.798	14.798	14.798	0.006
75	V_23_Plate1_B11	19	14.477	72.414	4.417	8.166	275.065	0.104
76	V_35_Plate1_C11	8	7.37	12.445	4.218	7.131	58.964	0.022
77	V_47_Plate1_D11	7	13.611	25.338	4.937	14.636	95.28	0.036
78	V_59_Plate1_E11	2	10.767	14.973	6.562	10.767	21.534	0.008
79	V_71_Plate1_F11	7	6.339	10.04	4.684	5.636	44.371	0.017
80	V_83_Plate1_G11	11	9.05	19.49	4.561	7.552	99.551	0.038
81	V_12_Plate1_A12	6	12.006	23.745	5.417	11.117	72.034	0.027
82	V_24_Plate1_B12	2	5.738	5.867	5.609	5.738	11.477	0.004

83	V_48_Plate1_D12	12	9.743	25.481	4.577	7.545	116.919	0.044
84	V_60_Plate1_E12	8	9.284	13.898	4.964	8.938	74.27	0.028
85	V_72_Plate1_F12	4	8.84	14.328	4.05	8.491	35.36	0.013
86	V_84_Plate1_G12	5	15.619	31.129	5.32	16.146	78.093	0.03
87	V_96_Plate1_H12	3	15.037	16.791	12.263	16.059	45.112	0.017
88	V_2_Plate2_A2	11	14.36	57.476	4.555	7.683	157.961	0.06
89	V_3_Plate2_A3	4	14.569	17.305	9.34	15.815	58.274	0.022

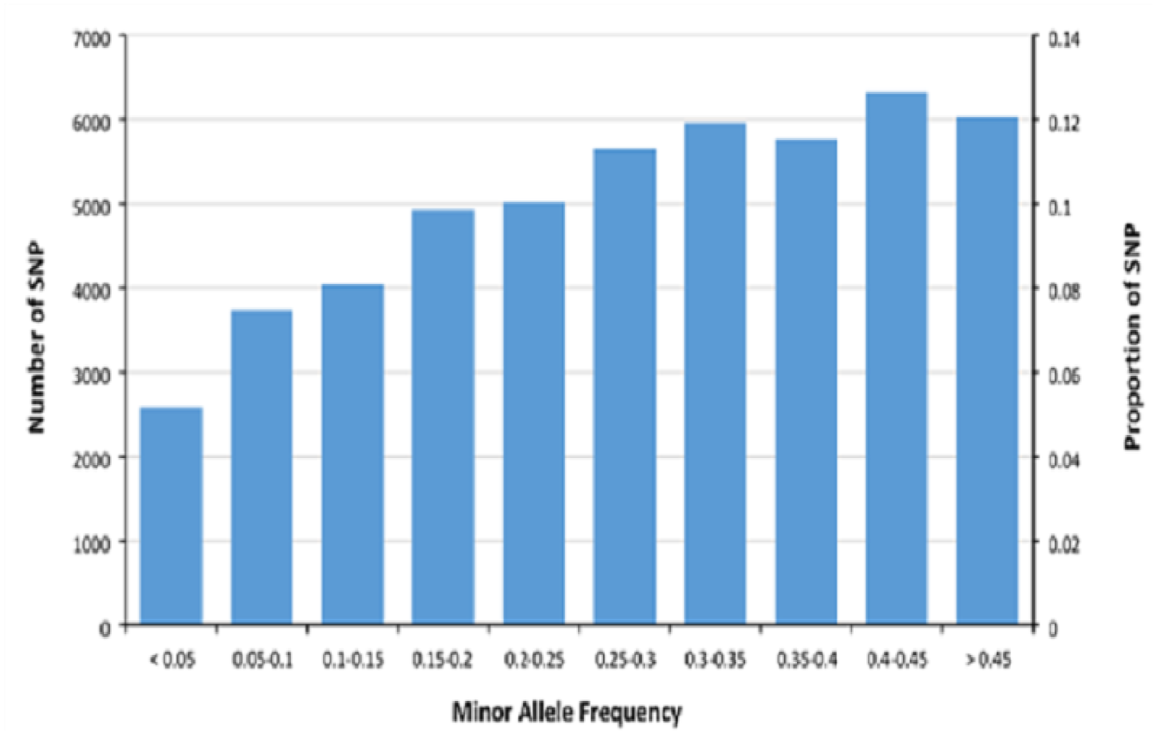

Fig. S1. Number and proportion of single-nucleotide polymorphism (SNP) markers with different minor-allele frequency (MAF). Approximately 48% of the SNP had MAF ≥0.30 and approximately 5.1% of the SNP had MAF between 0.01-0.05.

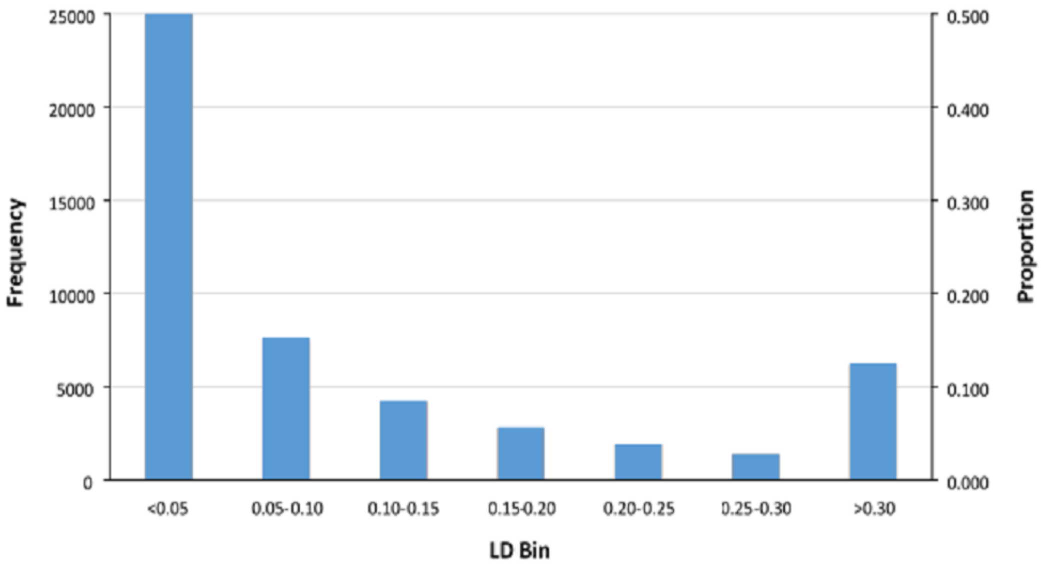

Fig. S2. Frequency and proportion of different pairwise r^2 values calculated for only the adjacent SNP pairs. The majority of adjacent SNP pairs (up to 51%) had $r^2<0.05$, and only 19.1% had $r^2>0.2$. Average r^2 of adjacent SNP pairs was 0.125±0.19.

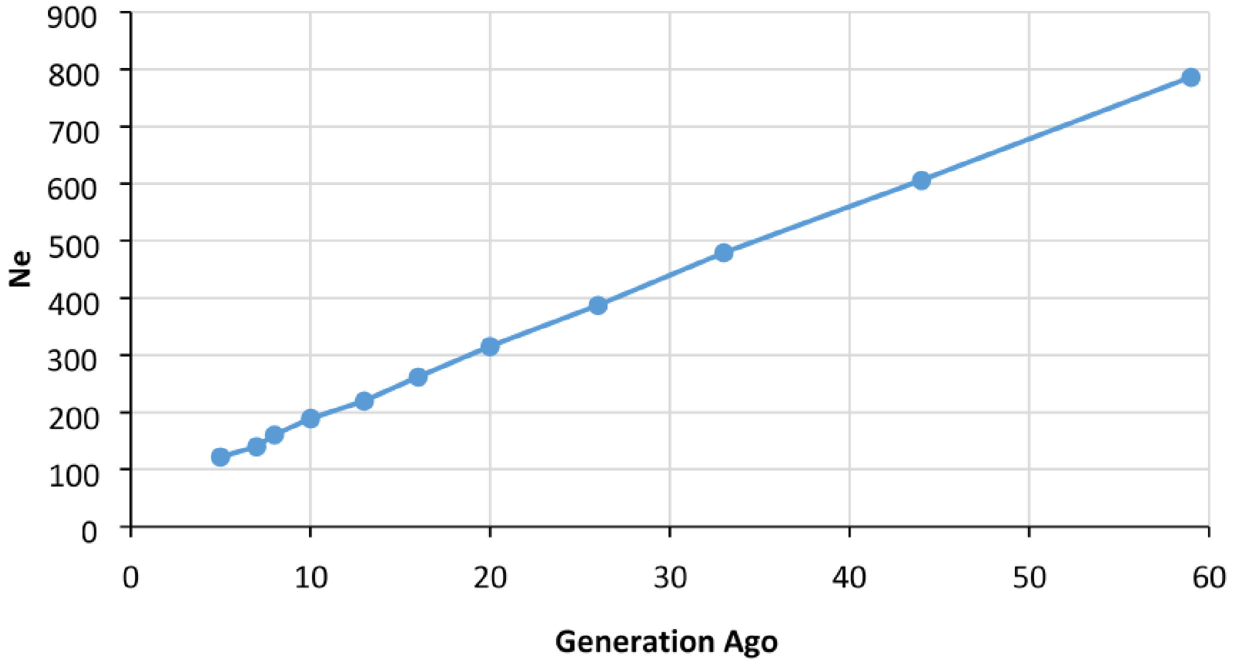

Fig. S3. Estimation of recent past effective population size (N_e) of Zandi sheep using genome-wide linkage disequilibrium (r^2) information. The estimated Ne of 59 and 5 generations ago were 786 and 122, respectively.
